# Microstructural and Mechanical-Property Manipulation through Rapid Dendrite Growth and Undercooling in an Fe-based Multinary Alloy

**DOI:** 10.1038/srep31684

**Published:** 2016-08-19

**Authors:** Ying Ruan, Amirhossein Mohajerani, Ming Dao

**Affiliations:** 1 MOE Key Laboratory of Space Applied Physics and Chemistry, Department of Applied Physics, Northwestern Polytechnical University, Xi’an 710072, PR China; 2 Department of Materials Science and Engineering, Massachusetts Institute of Technology, Cambridge, MA 02139, USA

## Abstract

Rapid dendrite growth in single- or dual-phase multicomponent alloys can be manipulated to improve the mechanical properties of such metallic materials. Rapid growth of (αFe) dendrites was realized in an undercooled Fe-5Ni-5Mo-5Ge-5Co (wt.%) multinary alloy using the glass fluxing method. The relationship between rapid dendrite growth and the micro-/nano-mechanical properties of the alloy was investigated by analyzing the grain refinement and microstructural evolution resulting from the rapid dendrite growth. It was found that (αFe) dendrites grow sluggishly within a low but wide undercooling range. Once the undercooling exceeds 250 K, the dendritic growth velocity increases steeply until reaching a plateau of 31.8 ms^−1^. The increase in the alloy Vickers microhardness with increasing dendritic growth velocity results from the hardening effects of increased grain/phase boundaries due to the grain refinement, the more homogeneous distribution of the second phase along the boundaries, and the more uniform distribution of solutes with increased contents inside the grain, as verified also by nanohardness maps. Once the dendritic growth velocity exceeds ~8 ms^−1^, the rate of Vickers microhardness increase slows down significantly with a further increase in dendritic growth velocity, owing to the microstructural transition of the (αFe) phase from a trunk-dendrite to an equiaxed-grain microstructure.

High temperature Fe-based alloys are attractive engineering materials that are currently used in a wide variety of industrial fields owing to their high performance, broad operational temperature range and low cost^1^,^2^,^3^,^4^,^5^,^6^. A solidification microstructure with refined grains and additional solute elements makes it possible to significantly improve their mechanical properties. Consequently, the relationship between the microstructure and the corresponding mechanical properties in multicomponent ferroalloys is a crucial scientific issue that requires systematic investigation.

Dendritic microstructure is the main microstructural constituent formed during the solidification process of single- or dual-phase Fe-based alloys. Rapid dendrite growth is realized by the rapid movement of the liquid/solid interface toward the undercooled melt. Consequently, the formation of rapidly grown dendrites is the result of a large deviation of the chemical equilibrium state at the solidification front. To simulate the rapid dendrite growth from the undercooled melt, marginal stability theory derived from Ivantsov equation^7^,^8^,^9^,^10^ and microscopic solvability theory considering interfacial free energy^11^,^12^,^13^,^14^,^15^ were successfully applied. However, these theoretical models are rarely used for multicomponent alloys because of the complicated interaction among solutes as well as the limited data available for the anisotropy parameter of the solid−liquid interfacial energy. In contrast, reliable experimental techniques have been developed to quantitatively measure the dendritic growth velocity as a function of the undercooling. Glass fluxing technique provides a feasible and straightforward approach to rapidly solidify alloys with a large undercooling and a low cooling rate. In this study, the bulk Fe-based alloy was prepared using the glass fluxing technique, and the rapid dendrite growth in the undercooled alloy was investigated in terms of the dendritic growth velocity.

Rapid dendrite growth results in grain refinement and microstructural evolution. Therefore, the influence of rapid dendrite growth on the mechanical properties of the alloy was mainly manifested in the grain refinement and microstructural changes versus altered mechanical properties. The grain refinement contribution to the improved mechanical properties of Fe-based alloys (especially for the hardness) is well established in the literature^16^,^17^,^18^. In the case of Fe-Ni alloy, Vickers microhardness as a function of grain size exhibits a well-defined Hall−Petch slope, whereas a gentler slope or even an inverse Hall−Petch behavior occurs once the grain size is reduced to the nanoscale^19^. To better understand the microstructure-based mechanical property changes related to the rapid dendrite growth, it is important to characterize the local mechanical property variations inside individual grains or across several refined grains. However, such knowledge has been lacking. Nanoindentation is a suitable approach for establishing such a mechanical property map corresponding to the microstructure; thus the correlation between the mechanical property and the microstructure can be elucidated. The nanoindentation technique can probe the mechanical properties with an applied force as low as the μN scale and the local displacements down to the nanometer scale. Over the past several decades, nanoindentation has been successfully employed to investigate the nanomechanical behavior of, e.g., thin films, porous structures, biological tissues and nanostructures^20^,^21^,^22^,^23^,^24^,^25^.

The main objective of this study is to elucidate the relationship between the rapid dendrite growth and mechanical properties of an Fe-based multicomponent alloy. Once we understand this correlation, the mechanical properties of metallic materials can be improved by controlling the dendritic growth velocity. In our work, the rapid dendrite growth of (αFe) from an undercooled Fe-based quinary alloy was realized first. The micromechanical properties of the alloys with increasing dendritic growth velocity were analyzed via Vickers microindentation. The local mechanical property variations of the solidified grains inside the alloys were also analyzed via nanoindentation.

## Results and Discussion

### Phase constitution and microstructures

**Figure 1:**
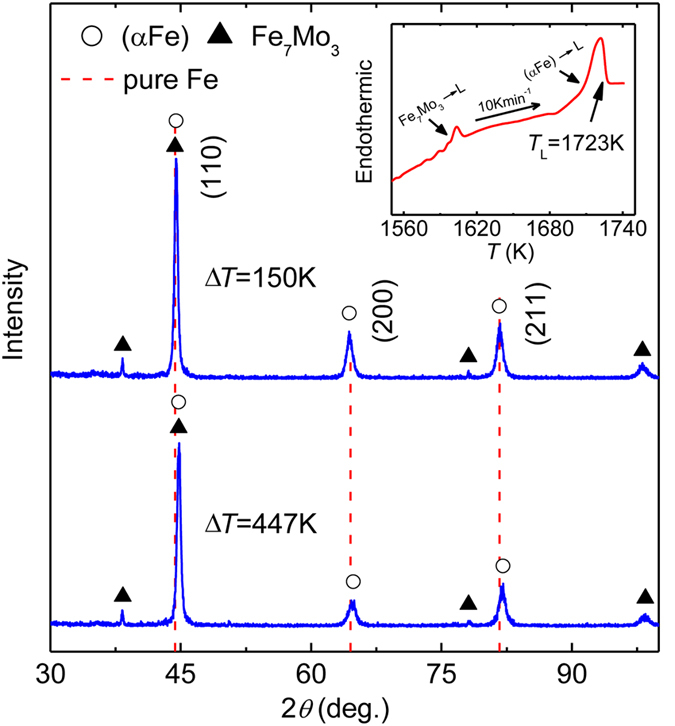
XRD patterns at the undercoolings of 150 K and 447 K. The inset is the DSC curve with the heating rate of 10 Kmin^−1^, and the liquidus temperature of the alloy is 1723 K as marked.

The liquidus temperature of Fe-5Ni-5Mo-5Ge-5Co alloy was determined by differential scanning calorimeter (DSC) analysis due to the lack of thermodynamic data for this alloy system. Two endothermic peaks were observed above 1560 K in the heating process, suggesting that two phase transitions may occur. Accordingly, the liquidus temperature of the alloy was determined to be 1723 K, as shown in the inset of Fig. 1.

The undercooling range of 67–447 K was obtained in the glass fluxing setup. XRD analysis was used for the alloy at different undercoolings to investigate phase constitution of the alloy and the influence of undercooling. Cu Kα radiation source was used at 40 kV and 200 mA. The XRD patterns of the alloys with the undercooling of 150 K and 447 K are plotted in Fig. 1. The undercooled alloy consists of the (αFe) solid solution and Fe_7_Mo_3_ intermetallic compound. We define the 2*θ* diffraction angle offset of the (αFe) solid solution as
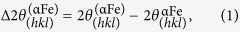
where 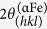 is the 2*θ* diffraction angle value for the (*hkl*) diffraction peak of the (αFe) solid solution, and 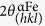 is the 2*θ* diffraction angle for the (*hkl*) diffraction peak of pure Fe (from JCPDS card 85–1410). The results are illustrated in Table 1. When the undercooling is low, there is only a minute 2*θ* angle offset between the diffraction peaks of (αFe) solid solution and the standard diffraction peaks of pure αFe. The 2*θ* angle values of the diffraction peaks increase slightly with the rise of the undercooling, reflecting the volume decrease of the unit cell of the (αFe) solid solution. This is caused by the variation of multi-solute contents in the (αFe) solid solution.

**Figure 2:**
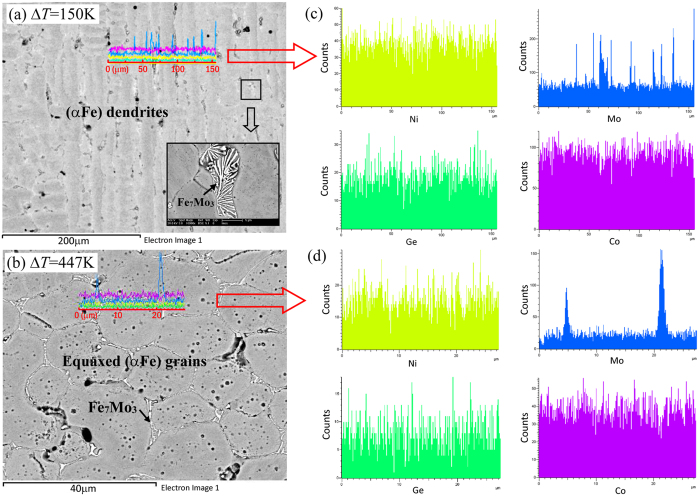
Microstructural morphologies and EDS line scan results of the undercooled alloys. (**a**) Δ*T* = 150 K; (**b**) Δ*T* = 447 K; (**c**,**d**) concentration profiles of solute elements across (αFe) second dendrite arms or equiaxed grains, the measurement locations are marked by the red lines in (**a**,**b**).

The typical microstructural morphologies of the alloy at different undercoolings are shown in Fig. 2. Based on observed microstructural characteristics of the alloy undercooled to different levels, we conclude that as the undercooling increases to ~290 K, the (αFe) dendrites with long trunks are transformed to the equiaxed grains. EDS scanning analysis was applied to verify the existence and distribution of the second phase. The EDS system was calibrated by using standard pure metals (all from Alfa Aesar, 99.999% pure). The measurement was carried out along two lines: a line across the second dendrite arms at the undercooling of 150 K; and a line across three equiaxed grains at the undercooling of 447 K, as shown in Fig. 2a,b. The Mo content increases dramatically either across the two adjacent secondary dendrite arms or on the equiaxed grain boundaries, indicating the presence of small Fe_7_Mo_3_ phase amounts. The lamellar interdendritic growth of the Fe_7_Mo_3_ phase is replaced by the refined homogeneous block growth of the Fe_7_Mo_3_ phase along the equiaxed grain boundaries. The detailed formation pattern of the second phase Fe_7_Mo_3_ is expected to influence the mechanical properties of the multicomponent alloy.

### Rapid growth behavior

Rapid growth of (αFe) dendrites is a crucial dynamic solidification behavior during rapid solidification of the undercooled alloy. The melting of (αFe) and Fe_7_Mo_3_ phases in the heating process of the alloy causes two endothermic events as revealed by the DSC measurement (see the inset of Fig. 1). Accordingly, experimental data for the (αFe) dendritic growth velocity were determined by detecting the first recalescence event during the solidification of the alloy.

**Figure 3:**
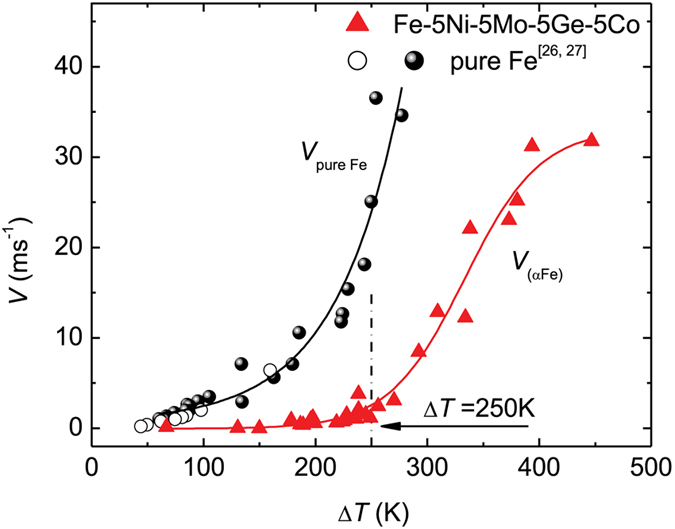
Experimentally measured growth velocities of (αFe) dendrites versus undercooling. In comparison, literature data for growth velocities of alpha dendrites in the pure component Fe measured under electromagnetic levitation condition are presented by hollow and solid circles^26^,^27^.

Figure 3 presents the (αFe) dendrite’s growth velocity, *V*_(αFe)_, as a function of the undercooling Δ*T*. As Δ*T* increases from 67 K to 250 K, the (αFe) dendrite’s growth velocity increases slowly to approximately 3 ms^−1^. When undercooling increases further, the growth velocity rises rapidly. Corresponding to the above mentioned critical undercooling of ~290 K, the critical dendritic growth velocity for the microstructural transition from dendrites with long trunks to equiaxed grains is ~8 ms^−1^, as seen in Fig. 3. Once the undercooling reaches 447 K, the growth velocity reaches the maximum value of 31.8 ms^−1^ and appears to be reaching a plateau. Compared with the alpha iron dendrites in the pure component Fe^26^,^27^, the alpha iron dendrites in the Fe-based multicomponent alloy require a larger undercooling to grow at the same rate (Fig. 3). In other words, the alpha iron dendrites in the multinary alloy require a larger dynamic driving force to grow than do those in the pure component.

According to the marginal stability theory, the essential parameters of dendritic growth (i.e., dendritic growth velocity *V* and dendrite tip radius *R*) are correlated with the undercooling Δ*T* in terms of thermal and chemical Peclét numbers, for which both the capillary and kinetic effects at the interface are ignored^7^,^8^,^9^. The relationship between the (αFe) dendritic growth and undercooling can be further discussed considering the non-equilibrium effect. Accordingly, the bulk undercooling is expressed as a sum of five terms^9^,^28^:
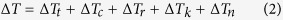
where Δ*T*_t_ is the thermal undercooling, Δ*T*_c_ is the solutal undercooling, Δ*T*_r_ is the curvature undercooling, Δ*T*_k_ is the kinetic undercooling, and Δ*T*_n_ is the undercooling caused by the shift of the equilibrium liquidus from its equilibrium position in the kinetic phase diagram of steady-state solidification. The solute contribution to the total undercooling is expressed by the solutal undercooling Δ*T*_c_. The calculation based on equation (2) for the Fe-based alloys indicates that the dendritic growth at low undercoolings is controlled predominantly by solute and thermal diffusion^28^,^29^. Indeed (αFe) dendrites were found to grow sluggishly within the undercooling range of 67 K < Δ*T* < 250 K in our experiment.

**Figure 4:**
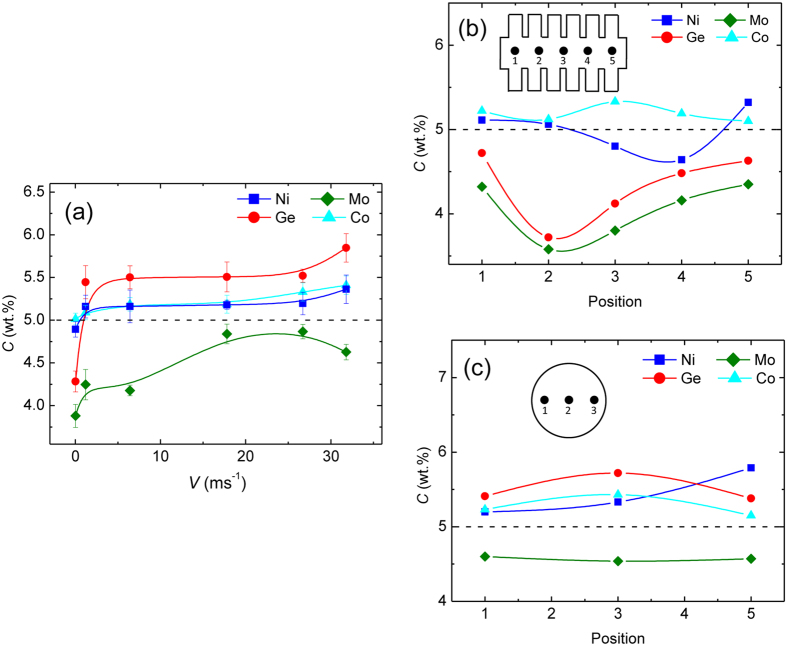
Solute contents of (αFe) dendrites: (**a**) average solute contents in the center of (αFe) dendrites versus dendritic growth velocity; (**b,c**) solute concentration profiles inside (αFe) grain at Δ*T* = 150 K and Δ*T* = 447 K respectively.

When the undercooling exceeds approximately 250 K, the growth velocity rises abruptly from nearly 3 ms^−1^ with a further increase in undercooling. This is likely related to the kinetic-controlled growth^28^ that is affected by both the composition gradient in the melt ahead of the interface and the composition change in the solid solution^30^,^31^. When the undercooling approaches 447 K, the growth velocity of (αFe) dendrites reaches a plateau. In the case of pure Ni and Ni-Cu alloy, such a dendritic-growth-velocity plateau is ascribed to the effect of residual oxygen that serves as an additional solute^32^,^33^,^34^. The experimental growth velocity plateaus in highly undercooled Ag and Ni-Si alloys can be well described by a diffusion-limited model compared with both LKT model^7^ and the collision-limited model used in molecular dynamics simulations^35^,^36^. In our experiments, the mechanisms of reaching the dendritic-growth-velocity plateaus are more complicated. Solute trapping also occurred, as confirmed by EDS analysis. Solute trapping occurring at a high undercooling was found to be accompanied by the transition from the ordered compound to the fully disordered compound in the Ni–Ge alloy^37^. In the case of Fe-based alloy, the occurrence of solute trapping inside (αFe) dendrites requires a large undercooling. Figure 4a shows the content variation of the four solutes in the center of an (αFe) grain with undercooling. The Ni, Ge and Co solutes’ contents increase with increasing dendritic growth velocity. In contrast, the Mo solute content first increases and then decreases slightly. Owing to the apparent Ge content increase, the unit cell volume of the (αFe) solid solution at the undercooling of 447 K decreases relative to that at the undercooling of 150 K, revealed by XRD analysis. Moreover, the distribution of all solutes inside an equiaxed grain becomes uniform with the maximum growth velocity, as shown in Fig. 4b,c.

### Influence of rapid dendrite growth on mechanical properties

**Figure 5:**
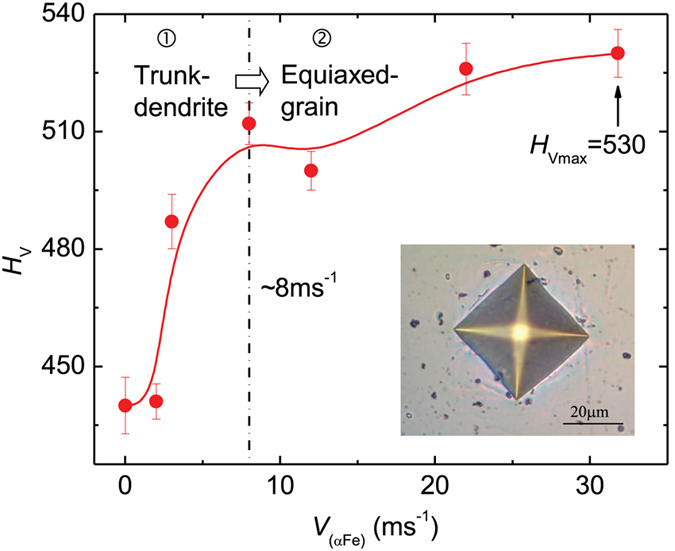
Dendritic growth velocity *V* versus Vickers microhardness *H*_V_. The inset is an image of a typical Vickers indentation performed in the sample.

As a crucial physical characteristic of solidification behavior, the growth velocity of (αFe) dendrites dramatically influences the mechanical properties of the alloy. Figure 5 shows the variation in Vickers microhardness, *H*_v_, as a function of the dendritic growth velocity, *V*_(αFe)_. *H*_v_ increases from 440 to 530 as *V*_(αFe)_ increases. The increasing trend of *H*_v_ with *V*_(αFe)_ can be divided into two stages: *H*_v_ increases steeply as *V*_(αFe)_ increases initially from 0.02 to ~8 ms^−1^, and it then increases slowly as *V*_(αFe)_ further increases to the maximum value of 31.8 ms^−1^.

Mechanical behavior changes are mainly attributed to the combined effects of grain refinement and microstructural evolution caused by the rapid dendrite growth. The influence on the mechanical properties is explained in terms of grain refinement and microstructural evolution in the following sections.

**Figure 6:**
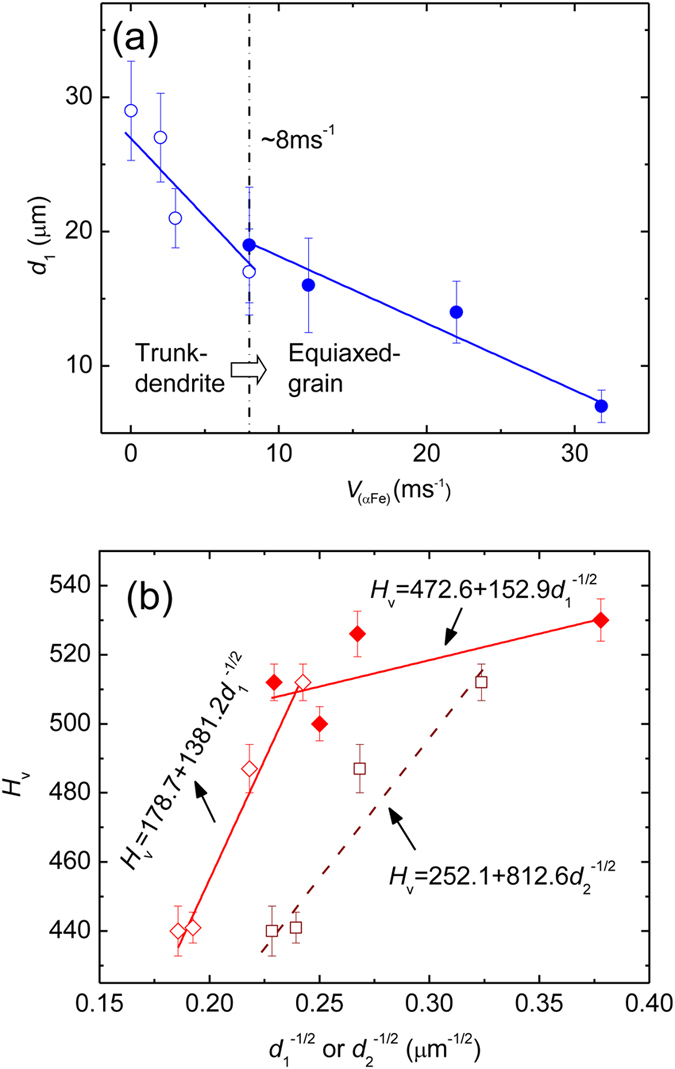
Influence of grain refinement on Vickers microhardness: (**a**) variation of the representative microstructural size *d*_1_ (represented by the dendrite width or equiaxed grain size) versus dendritic growth velocity *V*; (**b**) Hall−Petch plots in terms of *d*_1_ or secondary dendrite arm spacing *d*_2_. The hollow symbols show the trunk-dendrite data points and the solid symbols represent the equiaxed-grain values.

The dependence of the microhardness on dendritic growth velocity is directly related to the grain size variation caused by the rapid dendrite growth from the undercooled melt. Studies on some metal and alloys have noted that dendrite coarsening may occur during dendrite refining with the increase of undercooling^38^,^39^. In our experiment, (αFe) dendrites continuously refine with undercooling, and no dendrite coarsening phenomenon is found. Figure 6a shows the relationship between the average grain size and dendritic growth velocity. The nominal grain size *d*_1_ is defined as the (αFe) dendrite trunk width or equiaxed grain diameter. *d*_1_ of (αFe) dendrites with trunks decreases when *V*_(αFe)_ increases from 0.02 to 8 ms^−1^, whereas that of the equiaxed grain decreases with a slower pace with a further increase in *V*_(αFe)_. Consequently *H*_v_ first rises steeply and then increases slowly as *V*_(αFe)_ increases. Several grain refinement mechanisms have been proposed over the past years, including the copious nucleation ahead of the solidification front induced by a pressure pulse^40^, solute distribution^41^, recrystallization initiated by the stored deformation energy^42^, and dendrite fragmentation based on Karma’s model^43^. In a recent work^28^, it has been confirmed that dendrite fragmentation is the main cause for grain refinement in an Fe-based alloy.

Hall−Petch behavior is a well-known criterion for the relationship between the mechanical properties and grain size of alloys^44^,^45^,^46^,^47^. To directly explore the influence of grain refinement on microhardness, the Hall−Petch plot is shown in Fig. 6b, where *d*_1_ is the representative microstructural dimension (represented by the dendrite trunk width or the equiaxed-grain size). The slope of the curve is defined as Hall−Petch coefficient and decreases with decreasing dendrite trunk width or equiaxed grain size *d*_1_. The Hall−Petch behavior can be characterized by the two linear equations marked in Fig. 6b, as indicated by the two solid lines. If the representative microstructural size is represented by the secondary dendrite arm spacing *d*_2_, the corresponding Hall-Petch plot is also shown by the hollow squares and dashed line in Fig. 6b. The reduced Hall-Petch slope and reverse Hall−Petch behavior were reported previously when the grain size decreases to a critical value at the nanoscale (on the order of 10 nm), and they result from the transition from the intragranular to grain boundary mediated deformation^19^,^48^. A more recent study showed that cBN hardens continuously with decreasing grain size even down to the smallest nanosizes^49^. However, the reason for the bi-linear Hall−Petch behavior for micron-scaled grains in our experiments is different from that for the nanocrystalline materials. Based on the microstructural characteristics of the alloys shown in Fig. 2, the transition from dendrites with long trunks to equiaxed grains occurs with increasing dendritic growth velocity. The decrease of the Hall−Petch slope indicates that the Vickers microhardness increases much faster with the size reduction of the trunk-dendrite microstructure than with the size reduction of the equiaxed-grain microstructure. The secondary dendrite arms and the associated boundaries within the trunk-dendrite microstructure may also contribute to the fast microhardness increase.

The microstructural evolution caused by the rapid dendrite growth affects the mechanical properties as well. The relationship between the dendritic microstructure and mechanical properties at two dendritic growth velocities was investigated by establishing maps of mechanical property variations that corresponded to the changes in the microstructure. The nanoindentation experiment with over 660 indents was carried out. The data obtained from the indents are located on the phase boundaries or very close to them, or porosities were deleted to avoid artefacts. To avoid the data produced by local voids or roughness, we excluded the indents with extracted reduced elastic modulus *E*_r_ beyond ±12% of the average value 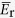. The average nanohardness values are higher than the average microhardness, which is attributed to the indentation size effect caused by the difference between the two experimental methods.

**Figure 7:**
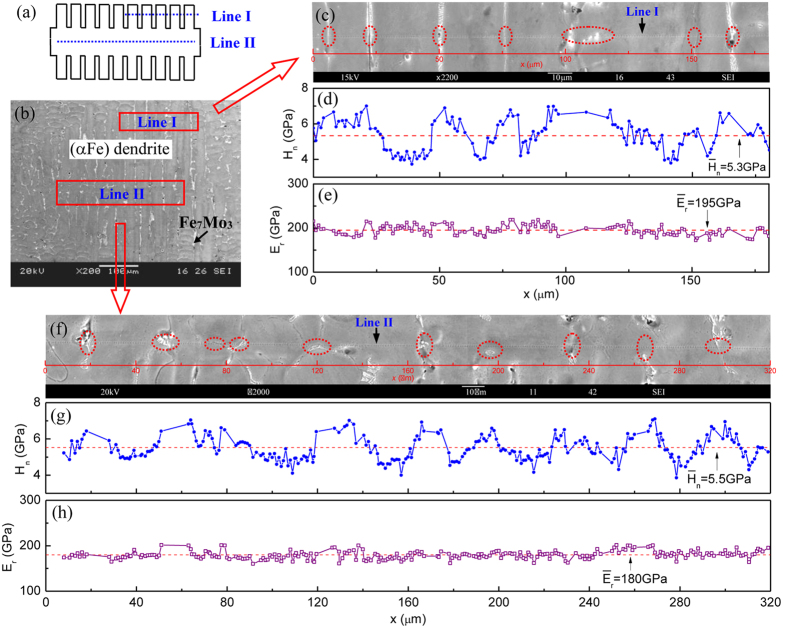
Nano-mechanical properties of the dendrites inside the alloy undercooled to 150 K with a dendritic growth velocity of 0.02 ms^−1^. The measurements were performed along two lines: across several secondary dendrite arms involving Fe_7_Mo_3_ phase and along the inner dendrite trunk. The two measurement locations represent the typical microstructures of the alloy: (**a,b**) schematic and image of the measurement locations marked by dotted lines; (**c–e**) variations of nanohardness and Young’s modulus across adjacent secondary dendrite arms; (**f**–**h**) variations of nanohardness and Young’s modulus along a dendrite trunk.

Figure 7 presents the maps of mechanical properties corresponding to the microstructure of the sample with the growth velocity of 0.02 ms^−1^ at a modest undercooling of 150 K. The nanohardness fluctuates considerably across a series of adjacent secondary dendrite arms with the maximum disparity of 3.3 GPa, as shown in Fig. 7d. Because of the distribution of the Fe_7_Mo_3_ phase along the grain boundaries and the hardening effect of the boundaries, the nanohardness on the sites between the secondary dendrite arms is evidently higher than that inside the dendrite. The nanohardness along the dendrite trunk is shown in Fig. 7g. It was difficult to expose the transverse section of the dendrite trunk accurately, and some indents are thus located on the grain/phase boundary. The nanohardness fluctuation also exists along the dendrite trunk where a very small amount of Fe_7_Mo_3_ phases exist nearby but the solutes are distributed inhomogenously. Based on Fig. 7d and g, it can be concluded that the nanohardness fluctuation is influenced by the following factors: the second phase, the grain/phase boundary, and the variation of multi-solute contents inside an (αFe) dendrite as confirmed by the multi-solute distribution shown in Fig. 4b.

Nanohardness is higher at the dendritic sites near the grain boundaries where the Fe_7_Mo_3_ phase exists than at the other dendritic sites. The hardening effect of the second phase and grain/phase boundary is well known in metallic systems^50^,^51^,^52^. The higher nanohardness at the sites near the grain boundaries is likely due to the combined effect of the grain boundary and Fe_7_Mo_3_ precipitation. A higher Mo content and the precipitation of the Fe_7_Mo_3_ phase were found to co-localize with the secondary dendrite arm boundaries (Fig. 2a,c). The hardening effect of the grain/phase boundary on the alloy may be linked to the well-known dislocations piled-up mechanism of the boundary^44^,^45^. In addition, the value of Young’s modulus along both lines I and II exhibits the fluctuations that are clearly smaller than those of the nanohardness. Table 2 shows the measured values and standard deviations of the alloy’s nanomechanical properties at the two dendritic growth velocities.

**Figure 8:**
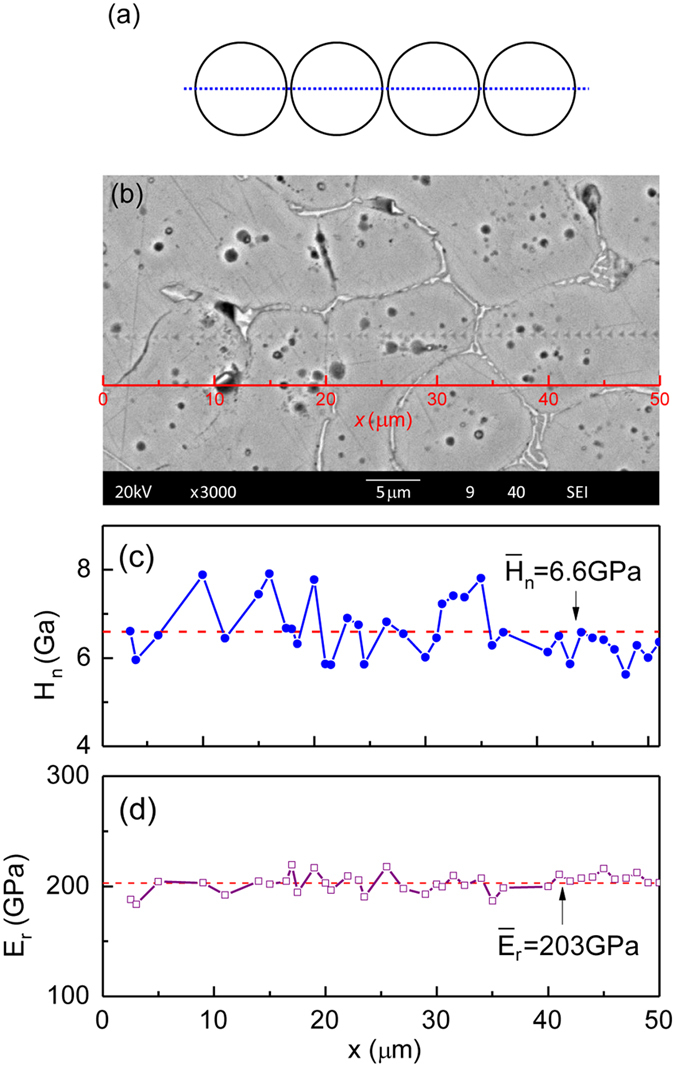
Nano-mechanical properties of equiaxed grains inside the alloy undercooled to 447K with a dendritic growth velocity of 31.8 ms^−1^: (**a,b**) schematic and image of the measurement locations marked by dotted line; (**c,d**) variations of nanohardness and Young’s modulus.

Figure 8 presents the nanohardness and Young’s modulus maps of the alloy at a high undercooling of 447 K with the maximum growth velocity of 31.8 ms^−1^. The measurements were performed along a line across a series of equiaxed grains. As dendritic growth velocity increases, the average nanohardness increases and becomes more homogeneous between the grains and inside a grain. Grain refinement caused by rapid dendrite growth results in a large number of grain boundaries and more homogeneous Fe_7_Mo_3_ phase distribution with smaller sizes; therefore, it hardens the alloy. Owing to the solute trapping occurring at high dendritic growth velocities, all of the solutes are distributed more uniformly inside the grain, and the solute contents increase (Fig. 4). This contributes to the homogeneous and improved mechanical properties. The difference between the nanohardness of the two phases can also be reduced by rapid solidification.

In summary, the mechanical properties of the multicomponent alloy can be improved by rapid dendrite growth, and the slope of *H*_V_*−V*_(αFe)_ curve decreases when the dendritic growth velocity becomes higher than ~8 ms^−1^. On the one hand, as the (αFe) dendritic growth velocity increases, (i) increased grain/phase boundaries serving as dislocation barriers caused by grain refinement, and (ii) a homogeneous distribution of both the second phase along the grain boundaries and the solutes inside the grains, both contribute to the increase of microhardness. On the other hand, the hardening effect from the refinement of the trunk-dendrites is stronger than that arising from the refinement of the equiaxed grains, therefore the rate of microhardness increase is reduced beyond the dendritic growth velocity of ~8 ms^−1^ owing to the microstructural evolution from the trunk-dendrite to the equiaxed-grain microstructure.

## Conclusions

The rapid growth of (αFe) dendrites was realized in the undercooled Fe-5Ni-5Mo-5Ge-5Co alloy by means of the glass fluxing method. The influence of rapid dendrite growth on micro- and nano-mechanical properties was investigated in this alloy. The key results and conclusions are summarized as follows:

1. The (αFe) dendrite’s growth velocity increases from 0.1 to 31.8 ms^−1^ with increasing undercooling. More undercooling is required for the rapid growth of alpha iron dendrites in the Fe-based multinary alloy than in the pure component Fe, corresponding to the complex changes in the kinetics and thermodynamics of dendrite growth in a multi-solute system. As a result, (αFe) dendrites grow sluggishly within a low but wide undercooling range. If the undercooling exceeds 250 K, (αFe) dendritic growth velocity increases steeply from 3 ms^−1^. When the undercooling reaches 447 K, the growth velocity tends to plateau.
2. The microstructure of the alloy consists of (αFe) dendrites and an interdendritic Fe_7_Mo_3_ phase. The (αFe) grains are refined with increasing undercooling. As the undercooling increases to ~290 K and the dendritic growth velocity increases to ~8 ms^−1^, the microstructure transforms from a trunk-dendrite to an equiaxed-grain microstructure. Additionally, solute trapping occurs at a high undercooling of 447 K as confirmed by EDS analysis, resulting in a homogeneous distribution of solutes and an increase of solute contents inside the (αFe) grains.
3. The increase of Vickers microhardness with increasing dendritic growth velocity is attributed to the increased grain/phase boundaries owing to grain refinement, the homogeneous distribution of the Fe_7_Mo_3_ phase along the boundaries and increased multi-solute contents inside the grains. Furthermore, Vickers microhardness increases first rapidly and then slowly beyond the dendritic growth velocity of ~8 ms^−1^. This is caused by the microstructural transition from a trunk-dendrite to an equiaxed-grain microstructure. The bi-linear Hall−Petch behavior suggests that the increase of the Vickers microhardness is more sensitive to the size reduction of the trunk-dendrite microstructure than to the size reduction of the equiaxed-grain microstructure. The maximum Vickers microhardness was found to be 530.
4. The hardening effects from the grain/phase boundary and the Fe_7_Mo_3_ phase were demonstrated directly by nanohardness maps corresponding to the microstructure. Nanohardness fluctuates either across the secondary dendrite arms or along the dendrite trunk. Compared to the nanohardness fluctuation in the dendrites, the fluctuation within the equiaxed grains is smaller, because of the more uniform distribution of the solutes with the increased solute contents caused by the solute trapping effect, and from the smaller difference between the nanohardness values of the two phases.

## Methods

### Rapid solidification of undercooled alloy

The Fe-5Ni-5Mo-5Ge-5Co (wt.%) master alloy was prepared by arc-melting of pure Fe (99.99%), pure Ni (99.999%), pure Mo (99.99%), pure Ge (99.999%) and pure Co (99.99%) in the protective ultrapure argon gas atmosphere. Each sample had a total mass of 1 g. The undercooling experiment was carried out using the glass fluxing method. The sample covered with designed denucleating agent (nominal chemical composition of 70%B_2_O_3_ + 20%Na_2_B_4_O_7_ + 10%CaF_2_) was placed in an alumina crucible in a vacuum chamber. The vacuum chamber was evacuated to a pressure of 2 × 10^−4^ Pa and refilled with ultrapure Ar gas. The sample was melted by RF (Radio Frequency) induction coil and cooled by adjusting the heating power. To achieve the intended undercooling, the sample was superheated to a temperature 100–300 K above its liquidus temperature and the heating-cooling process was repeated several times. During the experiment, the temperature was continuously monitored by a Yunnan-Land NQO8/15C infrared pyrometer calibrated by Pt-30Rh/Pt-6Rh thermocouples. The dendritic growth velocity was measured simultaneously by an infrared photodiode device. The as-solidified samples with mass loss less than 0.05% were cross-sectioned, mounted and polished. The liquidus temperature, phase constitution and microstructure of the prepared samples were analyzed using a NETZSCH STA 449 C DSC, a Rigaku D/max 2500V X-ray diffractometer (XRD), an Oxford INCA 300 electron dispersive spectrometer (EDS), an FEI Sirion 200 scanning electron microscope and a JEOL JSM-5910 (SEM), respectively.

### Measurement of mechanical properties

The Vickers microhardness of the undercooled alloy was measured using a LECO LM248AT Microhardness Tester. The applied load was 500 gf and the dwell time was kept constant at 15 s for all tests. After the load was removed, the two impression diagonals were measured with a micrometer eyepiece to obtain the average diagonal length. A standard specimen was tested to confirm that instrumental error is less than 1%. At least 30 valid indentations were made (only symmetric indentations were considered) in an area across the longitudinal section of each sample. To prevent the influence of the adjacent indents, the distance between two indentation points was more than three times the diagonal length of an indentation. Nanoindentation tests were performed using a Hysitron Tribo Indenter with the displacement and load resolutions of 0.1 nm and 0.1 μN, respectively. A diamond Berkovich probe, which is a three-sided pyramidal probe with the curvature radii of 150 nm, the included angle of 142.35° and the half-angle of 65.35°, was adopted for the test. The Berkovich tip was calibrated using a standard fused quartz sample. Three factors were considered in the design of our nanoindentation tests to ensure the accuracy of the experiments. First, sufficient nanoindentation points were chosen inside every grain to detect its continuous hardness change and to avoid inaccurate measurements related to the severe local inhomogeneity at the grain or phase boundaries. This type of error is due to the depth change at these sites or the pop-in effect from the grain boundary^53^. Second, the indentation was made reasonably deep to reduce the effects of the sample surface roughness^54^ and the size effect from nanoindentation method itself^55^. Third, the distance between the adjacent indentation points was assigned to be sufficiently large to prevent the indents from interfering with each other^56^. Based on these considerations, a series of 100 nm depth-controlled nanoindentations were carried out with an interval of 1 μm between the adjacent indents. The load function consists of loading up in 10 s, holding for 5 s at the peak load and unloading in 10 s in sequence. The Oliver−Pharr method^57^,^58^ was applied to calculate the nanohardness and Young’s modulus values, according to the experimental load–displacement curve.

## Additional Information

**How to cite this article**: Ruan, Y. *et al*. Microstructural and Mechanical-Property Manipulation through Rapid Dendrite Growth and Undercooling in an Fe-based Multinary Alloy. *Sci. Rep.* **6**, 31684; doi: 10.1038/srep31684 (2016).

## Figures and Tables

**Table 1:** Offset values of 2*θ* diffraction angles for (αFe) solid solution (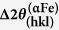) at different undercoolings.

Δ*T* (K)	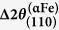 (deg.)	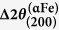 (deg.)	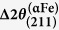 (deg.)
150	0.108	−0.146	0.086
447	0.468	0.414	0.446

**Table 2:** Nano-mechanical properties at different dendritic growth velocities. Δ*H*_n_ and Δ*E*_r_ represent standard deviations.

*V*_(αFe)_ (ms^−1^)	Location	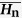 (GPa)	Δ*H*_n_	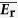 (GPa)	Δ*E*_r_
0.02	Line I: across secondary dendrite arms	5.3	0.9	195	11
0.02	LineII: along the dendrite trunk	5.5	0.7	180	9
31.8	Across equiaxed grains	6.6	0.6	203	9
